# Indigenous Telemedicine

**DOI:** 10.1016/s0972-6292(16)30504-6

**Published:** 2012-05-20

**Authors:** Neeraj Parakh, Vivek Chaturvedi

**Affiliations:** Department of Cardiology, G.B. Pant Hospital, New Delhi, India.

**Keywords:** Indigenous, Telemedicine

## Abstract

We are describing a simple and innovative way of documenting tachycardia. This patient came with video recording of neck pulsation done with the help of mobile phone camera. No other documentation of this tachycardia was available as patient was living in a remote area away from even basic health facilities.

## Case report

**Figure 1:**
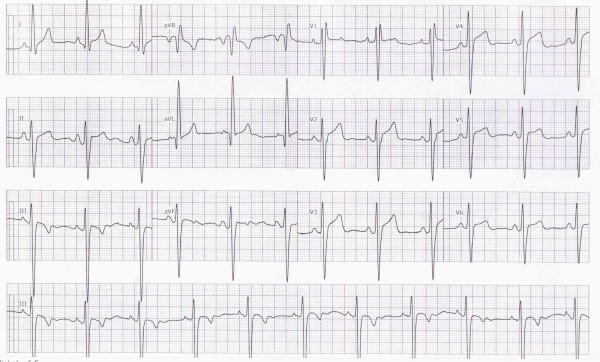
Baseline ECG in normal sinus rhythm showing RBBB with left axis deviation.

**Figure 2:**
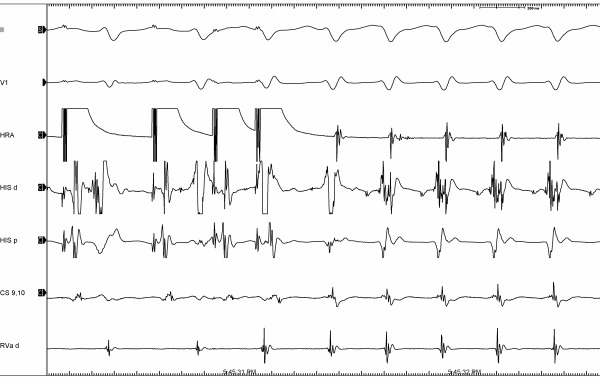
(A)EPS showing induction of tachycardia (on A pace and double extra systole) with AH jump and short VA interval.

It was during one of those bedside clinics, when one of my professors taught us that Wenckebach block was first diagnosed by Dr Karel F. Wenckebach in 1899 by observing jugular venous pulsation [1]. As a young doctor I always thought these people as super-humans who were able to make such diagnoses without the aid of even a simple electrocardiogram. A decade later, when I started practicing as a cardiac electrophysiologist, a little incident instantaneously brought forward these old memories. A young man entered in my clinic with his old mother. Even before they could take their seats the man started narrating his mother's problem. Apparently she was troubled by recurrent palpitations for last 15 years. These palpitations have become more troublesome for last 6 months. These palpitations were never recorded by means of an electrocardiogram as they lived in a remote village in one of the most backward states of India. Even when palpitations lasted for hours, lack of medical facilities and transport facilities in the area made it impossible for them to document the palpitations by a trained medical personnel or electrocardiogram (ECG). To my surprise, the son presented a video of neck pulsation recorded by his mobile phone camera (Video 1). A full two minute video that recorded neck pulsations from various angles and distances was played and the son watched me expectantly as if I will be able to take care of their problems then and there and all their trouble has come to an end. The neck pulsations were quiet rapid and regular and paroxysmal supraventricular tachycardia was my likely diagnosis. Still there was a big list of other differentials and in the absence of an ECG there was no way to tell them at that time that what is exactly happening. I just wondered what Dr Wenckebach would have done in this situation. Sinus rhythm ECG showed incomplete right bundle branch block and left axis deviation, without any evidence of pre-excitation (Figure 1). 24 hour Holter study failed to provide any further help as no tachycardia event happened during that period. This lady underwent electrophysiological study at a later date and that tachycardia turned out to be an atrioventricular nodal reentrant tachycardia (Figure 2). She was treated successfully by radiofrequency ablation of slow pathway.

In a country like India, where mobile phone industry has grown tremendously and made inroads into every nook and corners of the country, this kind of indigenous telemedicine is certainly a small filler in the big lacuna of health care.

## Figures and Tables

**Video 1:** Video of neck pulsations acquired from mobile phone camera: Click here to view the movie
